# Towards reliable isoform quantification using RNA-SEQ data

**DOI:** 10.1186/1471-2105-11-S3-S6

**Published:** 2010-04-29

**Authors:** Brian E Howard, Steffen Heber

**Affiliations:** 1 Bioinformatics Research Center, North Carolina State University, Raleigh, NC, 27606, USA

## Abstract

**Background:**

In eukaryotes, alternative splicing often generates multiple splice variants from a single gene. Here weexplore the use of RNA sequencing (RNA-Seq) datasets to address the isoform quantification problem. Given a set of known splice variants, the goal is to estimate the relative abundance of the individual variants.

**Methods:**

Our method employs a linear models framework to estimate the ratios of known isoforms in a sample. A key feature of our method is that it takes into account the non-uniformity of RNA-Seq read positions along the targeted transcripts.

**Results:**

Preliminary tests indicate that the model performs well on both simulated and real data. In two publicly available RNA-Seq datasets, we identified several alternatively-spliced genes with switch-like, on/off expression properties, as well as a number of other genes that varied more subtly in isoform expression. In many cases, genes exhibiting differential expression of alternatively spliced transcripts were not differentially expressed at the gene level.

**Conclusions:**

Given that changes in isoform expression level frequently involve a continuum of isoform ratios, rather than all-or-nothing expression, and that they are often independent of general gene expression changes, we anticipate that our research will contribute to revealing a so far uninvestigated layer of the transcriptome. We believe that, in the future, researchers will prioritize genes for functional analysis based not only on observed changes in gene expression levels, but also on changes in alternative splicing.

## Background

In higher organisms many multi-exon genes undergo alternative splicing (AS) reactions that produce multiple splice variants, often encoding distinct, but related, protein products. In contrast to the traditional “one gene, one protein” hypothesis, most, if not all, multi-exon genes are now believed to be subject to alternative splicing in human [1], with AS isoforms apparently responsible for many of the salient differences between diverse tissue types. A significant degree of AS has also been observed in various plant and animal species, although the precise magnitude and functional relevance of these events is unknown [2,3].

While it has long been assumed that differential gene splicing plays an important role in determining the phenotypes of organisms, it has been difficult to quantify AS using available high throughput methods. However, recently developed massively parallel sequencing-by-synthesis technologies from Illumina, Applied Biosystems and Roche 454 Life Sciences have the potential to revolutionize the study of the transcriptome [4]. It is now possible to produce enough high quality reads in a single run to rival traditional EST libraries that have accumulated over a span of decades. Furthermore, the resulting digital counts are more comparable to the “gold standard” quantification method, quantitative PCR (qPCR), and may overcome many of the shortcomings inherent in hybridization-based microarray gene expression studies.

Because the technological potential of these “RNA-Seq” protocols is well appreciated, and rapidly advancing, methods for accurate estimation of isoform expression levels are an active area of research. To compute an isoform expression score, the reads that map to each isoform must be converted into a quantitative expression value. One approach is to count the number of reads that map to each transcript, normalizing against the transcript length and sequencing depth [5]. Unfortunately, this technique is often infeasible for AS variants because many reads can map to multiple isoforms simultaneously. Recently, Lacroix *et al.* investigated the theoretical limitations of transcriptome reconstruction and quantification from a combinatorial perspective [6]. Their analysis operated under an “exact information hypothesis” whereby the exact abundances of all relevant transcribed regions is provided error-free. However, this approach ignores the sampling process that actually generates observed data along with the associated measurement error; in practice, statistical approaches are necessary in order to obtain accurate estimates of transcript abundance. For example, Jiang and Wong have described a Poisson model for isoform quantification, showing how to estimate its parameters with a maximum likelihood approach [7]. Other authors have employed more basic (but effective) statistical approaches, for example, Fisher’s exact test, to compare levels of AS between treatments, e.g. [1].

In this paper, we explore the use of RNA-Seq datasets to address the “isoform expression estimation problem” as defined in [7]. It is assumed that the set of splice variants is known; the goal is to estimate the relative expression levels of these isoforms in a mixture. Obtaining precise estimates is necessary because important tissue-specific differences in AS frequently involve a continuum of isoform ratios, rather than all-or-nothing expression [8]. Although, currently, the assumption of known isoforms may be limiting in many cases, we will soon be able to construct detailed lists of known isoforms for various organisms and tissue states using high-throughput sequencing (see for example, [9]). A key advantage of our method over prior approaches is that our model takes into account the non-uniformity of RNA-Seq reads along the targeted transcripts. In addition, our approach can be easily adapted for use with any high-throughput sequencing technology, including those that employ paired reads. In the following sections we will describe the details of our model, demonstrate its performance on simulated and real data, and outline topics for future research.

## Methods

### Model overview

Given a set of *n* unique AS isoforms for a gene, *g*, it is always possible to partition RNA-Seq reads from *g* into 2*^n^* categories according to what subset of these isoforms each read is compatible with. For example, consider two AS isoforms, T_1_ and T_2_:

T_1_: AAAAAAA UUUUUUUUUU CCCCCCCCCC

T_2_: AAAAAAA ---------- CCCCCCCCCC

In this example, transcript T_1_ contains a cassette exon containing only “U” nucleotides. Transcript T_2_ skips this exon. Reads aligned to these transcripts can be classified into 3 mutually-exclusive subsets:

• *Subset S_1_*: Reads which contain U’s are only compatible with transcript isoform T_1_.

• *Subset S_2_*: Reads which contain A’s followed immediately by C’s (e.g. AAACCCCC) are only compatible with T_2_.

• *Subset S_3_*: Reads which contain only A’s or only C’s are compatible with both T_1_ and T_2_.

In addition, many reads, including reads containing one or more G’s, are not compatible with either T_1_or T_2_, but in the following we will disregard these and only consider reads that map to at least one of the known isoforms. Let:

Pr(S_i_) 	denote the probability that one of the gene’s reads maps to subset S_i_

Pr(T_i_) 	denote the probability that one of the gene’s reads maps to transcript T_i_

*Ø*_i_ 	denote the percentage of the gene’s transcripts expressed as isoform T_i_

Given the subsets introduced above, the following equation describes the probability that an individual read maps to subset S_i_:

 In general, we can assume that , the probability an individual read maps to a particular transcript, is dependent on the (unknown) frequency, *Ø*_j_, of that transcript in the transcript mixture. We will also assume that a given isoform is sampled with probability proportional to its known length. Similarly, , the probability that an individual read maps to subset S_i_, given the read maps to transcript T_j_, can be worked out using the known transcript sequence and estimates of the distributions for read length and read start position (for details, see the section “Constructing the Design Matrix”). Let:

*Y_i_* denote the number of reads compatible with subset S_i_

*R* denote the total number of reads for the gene

*p_ij_* = 

 = 

Assuming that individual reads are independent and identically distributed (iid), we then have *Y_i_* ~ Binomial( *R*,  ), and

For the example shown above, we can express this linear model in matrix form as follows:

Because *Rp*_21_ and *Rp*_12_ will always be zero, the rank of this matrix is 2, and both *β*_1_ and *β*_2_ are estimable.  Although, in general, the number of rows (2*^n^* – 1) grows exponentially with the number of possible transcripts, it is possible to either combine or ignore uninteresting categories. In fact, a full rank design matrix can always be constructed by considering only the *n* subsets consisting of reads that map to a single isoform.

### Distribution of read start position and read length

Most methods for estimating isoform abundance assume a uniform sampling distribution for reads along the targeted transcripts (e.g. [5-7]). However, it is widely acknowledged that the true distribution for read position deviates substantially from uniformity, and varies with the fragmentation protocol and sequencing technology [4]. Consequently, accurate methods for isoform quantification must incorporate this critical information.

**Figure 1:**
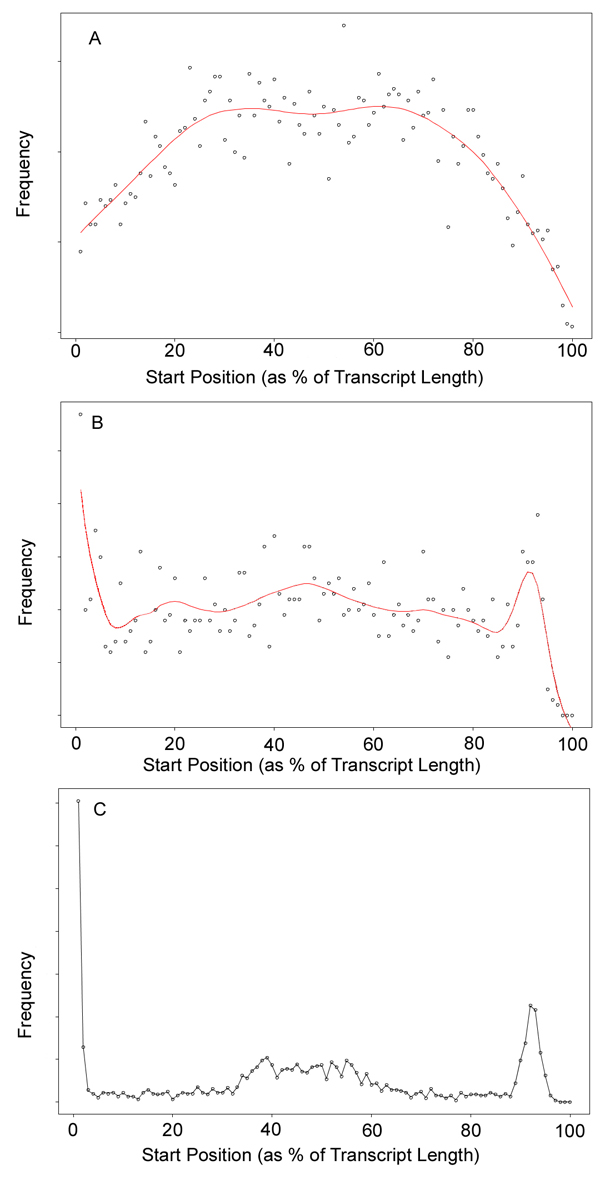
**Distribution of read start position as a percentage length for genes with median length of 1200 nucleotides.** The red line is a cubic spline fit. (A) Illumina dataset - RNA fragmentation by sonication [10] (dataset 1), (B) Roche 454 dataset - cDNA fragmentation by nebulization [14], and (C) Simulated cDNA fragmentation assuming fragments in the size range of 500-800 nucleotides.

We believe that these distributions should be consistent properties of the instrument and experimental protocol. With millions of reads often available per experiment, it is feasible to determine these distributions with a high level of accuracy. We used a kernel density approach to estimate read length and read start distributions using the observed empirical distributions observed for well-annotated transcripts (Figure 1a, 1b). The read length was estimated in a similar manner, resulting in an average read length of approximately 30 nucleotides for the Illumina data set, and about 100 nucleotides for the 454 dataset.

To investigate the relationship between experimental protocol and read distribution, we also created a simple simulation that emulates the process of cDNA fragmentation by nebulization. The similarity between Figure 1b and Figure 1c suggests that our simulation captures the main properties of the nebulization process. We anticipate that more detailed models, which incorporate knowledge of the physical processes of fragmentation and sequencing, should be able to describe observed distributions of read length and position even more accurately.

### Constructing the design matrix

**Figure 2:**
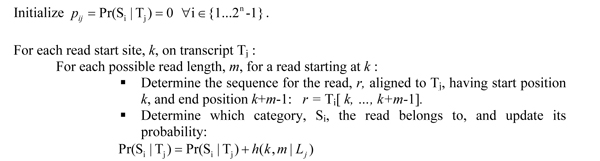
Procedure for computing the design matrix.

Let *h*( *k*, *m* | *L* ) denote the bivariate probability mass function describing the probability that a read has start position *k* and length *m*, given that this read aligns to a transcript of length *L*. We compute  for a particular transcript T_j_ using the procedure detailed in Figure 2.

### Estimation of *β* and *Φ*

Given the construction method described above, the design matrix will always be full column rank, so  will always be fully estimable. Each *Y_i_* ~ Binomial(*R*, ). For computational simplicity, we use the Normal approximation to the binomial distribution. For the Normal linear model with a known covariance matrix, the maximum likelihood estimate (MLE) obtained using weighted least squares is the best linear unbiased estimator (BLUE). In the system described above, the variances are not known, but can be estimated from the data. In this case, the “feasible weighted least squares” method can be used to approximate the weighted least squares solution. In cases where a resulting  is not a valid probability, we truncate the estimate at 0 or 1. In addition, we ensure that the total probability is one by dividing each   by the sum across all *i*.

Under the commonly employed assumption that a given isoform is sampled with probability proportional to its length, the probability that a given read maps to a transcript isoform can be expressed as follows:

where *L_j_* is the length of transcript *j* in bases. Given our estimates for the *β_j_* ’s, the known lengths for each transcript, and the fact that  , the  are uniquely determined and can be obtained by computing the unique  solution  to a  set  of  linear  equations. The sampling distribution of the resulting  cannot easily be computed analytically, but confidence intervals can be worked out empirically following a procedure based on [10].

### Multireads

An important consideration when dealing with short length RNA-Seq reads is that many reads can map to more than one gene. For example, using 25 nucleotide Illumina reads, Mortazavi, *et al.* found that between 13-25% of the reads were “multireads,” matching multiple genes in the mouse genome [5].

Two obvious extensions to the procedure described above can be made to accommodate multireads. One approach is to identify sets of genes and transcripts that share overlapping regions and jointly estimate the abundances of all transcripts in the set. However, since the size of the design matrix increases rapidly with the number of transcripts jointly estimated, this method may not be ideal when the number of overlapping genes is large.

A second approach is to discard those reads that map to more than one gene, after making an appropriate adjustment to the design matrix. When computing the probabilities as described in Figure 2, each potential read for a given transcript is checked against all other genes in the target dataset. If the read does not map to any other genes, the probability  is updated as described in Figure 2; however, if the read is a multiread, the read is skipped without updating . This test can be performed efficiently after pre-computing a hash table mapping nucleotide *k*-mers (e.g. *k* = 25) to genes which contain that sequence in one or more transcript isoforms. Given this modification to the probabilities in the design matrix, observed reads that map to more than one gene can now be discarded.

In the following research, we employ the first approach for the purpose of simulation and data analysis.

### Datasets

We tested the algorithm on two publicly available datasets. Dataset 1 (NCBI Short Read Archive experiments SRX002554 and SRX002555 [11]) consists of two experiments containing approximately 65 million (SRX002554, excluding run SRR013412, which was not available at the time of the analysis) and 57 million (SRX002555) Illumina reads from floral tissue from two different Arabidopsis strains (SRX002554 = col-0; SRX002555 = ddc). Dataset 2 (NCBI Short Read Archive Experiments SRX006704 and SRX006688 [12]) includes approximately 43 million (SRX006704) and 35 million (SRX006688) Illumina reads from control and drought-stressed Arabidopsis plant tissue.

### Implementation

The algorithm described above was implemented in Java, with matrix computations by the JAMA matrix library (available at http://math.nist.gov/javanumerics/jama/). Data analyses and simulations were also performed using the R statistical programming language (http://www.r-project.org/).

## Results

### Simulation

**Figure 3:**
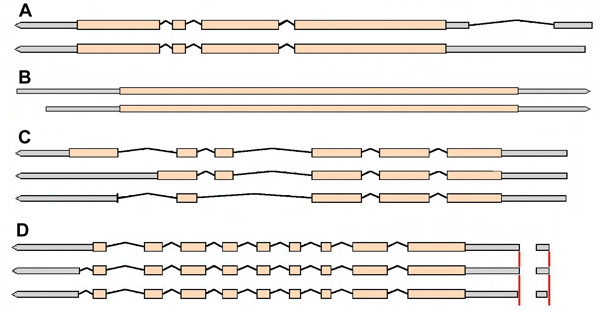
**Example of several TAIR 8 gene models used in alternative splicing simulation.** (A) AT1G75410, (B) AT2G40140, (C) AT2G01260 and (D) AT1G75380.

To test our method, we performed a simulated RNA-Seq experiment using the Arabidopsis gene models defined in TAIR 8 (http://www.arabidopsis.org) [13]. A publicly available dataset (dataset 1 in Methods) was used to estimate the read length and read start position for Illumina reads (Figure 1a). The simulation was performed for several of the multi-isoform genes as follows: First, a relative frequency for each of the alternative isoforms was specified as a simulation parameter, along with a predetermined total number of RNA-Seq reads. Each of these reads was then simulated by first selecting an isoform with probability proportional to its length and concentration in the mixture. Then, a read start position *k* and read length *m* along the selected isoform were drawn from the distribution, *h*( *k*, *m* | *L* ). Using these read coordinates, the nucleotide sequence along the sampled isoform was determined, and this sequence was compared with other isoforms in the mixture to identify which subset of isoforms the read is compatible with. The output of one run of the simulation is a list of subsets and the corresponding counts of simulated reads assigned to those subsets. For each gene, the entire simulation was repeated 500 times. Given the simulated datasets, we then used the linear model described above to infer the original isoform concentrations from the simulated subset counts. We performed the estimation in two different ways for each gene: first, using a design matrix constructed using the same read position distribution used to generate the simulated reads (Figure 1a) and second, using a design matrix constructed from a uniform read position distribution. Note that using the incorrect distribution can introduce a severe bias into the estimates and even change the ordering of the isoform expression levels (e.g. Table 1, row d; Figure 3d).

**Table 1:** Estimates for phi using 2000 simulated reads

	Transcript Mixture (*Ø*_i_)	**- True Read Distribution**	**- Uniform Read Distribution**
**A)**	AT1G75410.1=**70%**	**69.8%** (64.2%-75.2%)	**67.5% **(61.5%-73.3%)
	AT1G75410.2=**30%**	**30.2%** (24.8%-35.8%)	**32.5%** (26.7%-38.5%)

**B)**	AT2G40140.1=**70%**	**70.0% **(55.8%-83.9%)	**55.5%** (43.9%-68.4%)
	AT2G40140.2=**30%**	**30.0%** (16.1%-44.2%)	**44.5%** (31.6%-56.1%)

**C)**	AT2G01260.1=**20%**	**19.6%** (08.7%-30.5%)	**12.8%** (01.0%-23.9%)
	AT2G01260.2=**70%**	**70.3%** (62.5%-77.8%)	**76.4%** (68.5%-83.8%)

	AT2G01260.3=**10%**	**09.9%** (02.2%-18.8%)	**10.7%** (02.4%-19.9%)
**D)**	AT1G75380.1=**70%**	**69.5%** (58.9%-78.8%)	**70.9%** (60.8%-80.3%)
	AT1G75380.2=**20%**	**20.7%** (05.9%-33.9%)	**04.4%** (00.0%-21.4%)
	AT1G75380.3=**10%**	**09.4%** (0.00%-22.4%)	**22.9%** (09.3%-35.7%)

For each of the 500 replications, we also computed approximate confidence intervals about the estimates. To evaluate the performance of our approximation, we checked each confidence interval to see if it included the true value for the parameter. For the AT1G75410 simulation, 97.4% of the 95% C.I.’s contained the true parameter; 92.2% of the 90% C.I.’s contained the true parameter; and 63.2% of the 65% C.I.’s contained the true parameter.

This entire process was repeated for 100 different genes. Over several replications, the mean estimates were always approximately equal to the simulated isoform concentrations when the correct distribution was used to construct the design matrix. For genes with two isoforms, we found that approximately 750-1000 reads were often needed to obtain a 95% confidence interval with a width of ~20%. However, the number of reads required varies according to mixture composition, number of isoforms, and the read length and start site distributions. (Data not shown).

### Real RNA-Seq datasets

We next tested our method on two publicly available RNA-Seq datasets (see Methods). For each dataset, reads were mapped to the transcriptome using the SOAP v2 alignment program [15]. TAIR 8 was used to define the tested gene models. Among the 33282 Arabidopsis gene models defined in TAIR 8, 4330 genes had more than 1 isoform. In particular, 3336 genes had 2 isoforms, 739 had 3 isoforms, 186 had 4 isoforms, 48 had 5 isoforms, 14 had 6 isoforms, 4 had 7 isoforms, 2 had 8 isoforms, and 1 had 10 isoforms.

For dataset 1, in the col-0 sample (SRX002554 ), among genes with 2 or more isoforms, 1039 genes had more than 500 mapped reads (31.1%); 481 genes had more than 1000 reads (11.1%); 205 genes had more than 2000 reads ( 4.7%); and 50 genes had more than 5000 reads ( 1.5%). Similar coverage was observed in the other samples.

### Differential splicing

We first used a chi-square test of subset counts to identify genes that were differentially spliced between the two conditions. Genes that did not have at least one read mapping to the unique regions of at least two isoforms were not tested. In dataset 1, 305 of 1340 tested multi-isoform genes (22.8%) showed significant differential splicing between the two treatments. Significance was determined using a p-value cut-off of approximately 0.002, corresponding to a false discovery rate of 0.01 [16]. In dataset 2, 169 of 1705 tested multi-isoform genes (9.9%) showed differential splicing, according to the same criteria.

**Figure 4:**
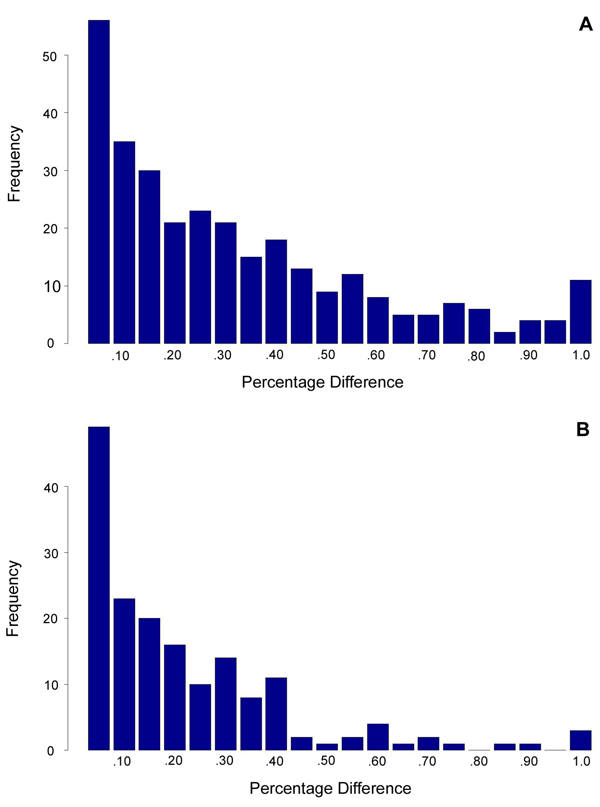
**Differences in main isoform frequency for AS genes.** Some of the changes are small and may not be biologically significant. Other genes show apparent “switch-like” regulation. (A) dataset 1, (B) dataset 2.

After identifying differentially spliced genes, we then used the linear model described above to infer the isoform ratios within each of the treatment samples. We discarded reads that mapped to more than one gene, using the adjusted design matrices computed as described in the Methods section. In several cases, genes with highly significant AS levels (according to the chi-square test), had only very small differences in isoform composition between the two treatments. These most likely do not represent significant biological differences. On the other hand, for both datasets 1 and 2, we also identified many genes that had large differences in isoform composition between the two treatments. In dataset 1, for example, 184 of the 305 genes (60%) had differences of 15% or more in the proportion for the main isoform; similarly, in dataset 2, 77 of 169 genes (46%) showed a 15% or greater difference in the proportion for the main isoform. Figure 4 shows histograms illustrating the distribution of differences in main isoform proportions between the two treatments within the two datasets. In particular, several genes appear to exhibit on/off “switch-like” differential splicing (shown in Figure 4 as genes with large differences in isoform expression between treatments). Full listings of the differentially spliced genes identified in each dataset, along with expression level estimates for the main isoform, can be found in Additional files 1 and 2.

To further characterize the genes identified as exhibiting differential splicing within the two datasets, we performed a GO analysis [17] using the online agriGO tool [18]. The program takes as input a list of genes and compares their associated GO terms with those corresponding to a relevant background set. Enriched terms are identified and grouped according to the GO categories “cellular component,” “biological process,” and “molecular function.” To perform the analysis, we submitted the lists of differentially spliced genes, and selected the Arabidopsis TAIR gene models as the background reference. We used the hypergeometric statistical test option, with the Yekutieli FDR multi-test adjustment method, set at a significance level of 0.10.

 For dataset 1, which compares leaf tissue for two different untreated strains of Arabidopsis, most of the significant GO terms were in the cellular component category GO category. Notably, many genes were associated with terms identifying cell membrane and membrane-bound cellular components, and the GO terms “membrane,” “plasma membrane,” “thylakoid membrane,” and “membrane-bounded organelle,” were all significant. This is of interest since it has been previously shown that AS is often used as a mechanism to alter the functional domains of transmembrane proteins [19-21]. In the molecular function category, the AS gene list was enriched for the terms “structural molecule activity”, “structural constituent of ribosome”, “nucleic acid binding” and “ligase activity”. In the biological process category, enriched terms include “developmental process”, “multicellular organismal development,” and “post-embryonic development”. A full list of the significant GO terms is included in Additional file 1, and graphical representations of the hierarchy of significant terms in the three main GO categories are available in Additional files 3, 4, 5.

**Figure 5:**
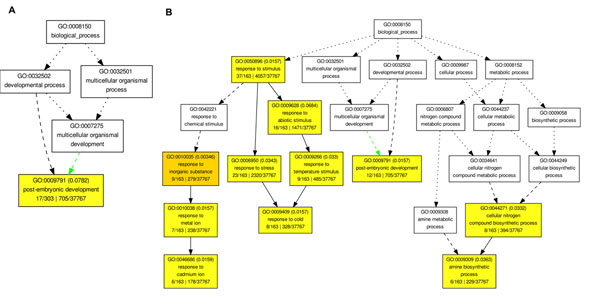
**Enriched terms under the “biological process” GO category for differentially spliced genes.** In dataset 2, which compares drought stressed plants to untreated controls, many of the significant terms are related to stress and stimulus response (B). In dataset 1, which compares two untreated strains of Arabidopsis, the enriched terms are more generic (A).

In dataset 2, which compares drought-stressed Arabidopsis plants to untreated controls, the list of differentially spliced genes was significantly enriched for many terms in the biological process category related to stress response, including “response to stress”, “response to stimulus”, “response to cold,” “response to chemical stimulus,” “response to cadmium ion”, etc. The significant biological process GO terms for both datasets are shown in Figure 5. Enriched cellular component GO terms for dataset 2 include “membrane-bounded organelle”, “cytoplasm”, and “ribosomal subunit,” among others. The full lists are available in Additional file 2, with graphical representations of the significant terms available in Additional files 6 and 7.

### Differential gene expression

For each dataset, we also tested for differential gene expression between the two treatments (independent of isoform composition). For each gene, we computed, within each treatment, *i*, the proportion of reads for that gene,, relative to the total number of reads in the sample, *n_i_*. The resulting proportions for each treatment were then compared on a gene-by-gene basis using the statistic  where. Since *n_1_* and *n_2_* are both very large, and the samples are independent, the test statistic, *z*, is assumed to follow a standard normal distribution. Because our goal was to compare differentially expressed genes with differentially spliced genes, only genes expressing more than one isoform were considered (1340 genes in dataset 1, and 1705 in dataset 2). The resulting p-values were subsequently corrected for multiple testing using the Benjamini-Hochberg method, with a FDR of 0.01 [16].

**Figure 6:**
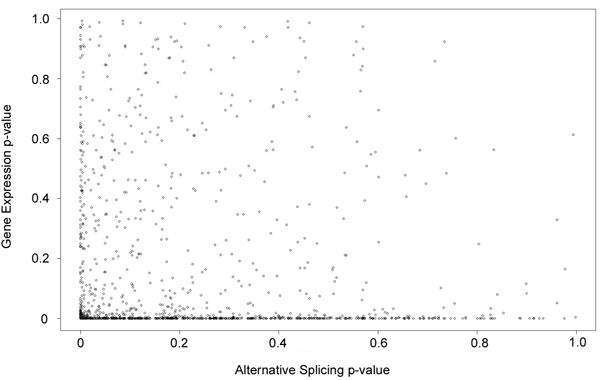
**Scatter plot of p-values for tests for alternative splicing and differential expression in dataset 1.** The correlation is -0.03, suggesting that differential gene expression and differential splicing may be independently regulated.

 In dataset 1, 58.6% of the tested genes showed differential expression between treatments according to these criteria; in dataset 2, 51.4% of the genes showed differential expression. In concordance with similar observations for animal systems [3], differential gene expression and differential splicing events appeared to be statistically independent. In dataset 1, for example, since 58.6% of the 1340 multi-isoform genes were differentially expressed, one would expect a random selection from those genes to include, on average, about 58.6% differentially expressed genes. Accordingly, the set of 305 differentially spliced genes included 180 differentially expressed genes (59.0%). In dataset 2, 118 (69.8%) of the differentially spliced genes were also differentially expressed at the gene level. (See Additional files 1 and 2 for details). The correlation between AS and differential gene expression p-values was -0.03 for dataset 1 (Figure 6) and 0.06 for dataset 2.

### Multireads and connected components

In dataset 1, 23.3% of the reads mapped to more than one gene; similarly, 27.5% of the reads were multireads in dataset 2. In the analysis described above, these reads were discarded according to procedure outlined in Methods, after an appropriate adjustment to the design matrix. An alternative technique for accommodating multireads is to jointly estimate all transcripts that share common read-length subsequences. To determine what genes should be jointly estimated, and to assess the feasibility of this approach for the Arabidopsis genome, we constructed a graph with TAIR 8 gene models as the vertices. In the graph, two vertices are connected with an (undirected) edge if their corresponding gene transcripts contain a common 25 nucleotide substring. The graph was then partitioned into its connected components. A connected component is a subgraph containing a set of vertices that meet the following conditions: 1) each vertex in the set can reach any other vertex in the set along some path in the graph, and 2) each vertex can not reach any vertex outside the set. Thus, the connected components partition the graph into sets of genes that should be jointly estimated using a shared design matrix.

**Figure 7:**
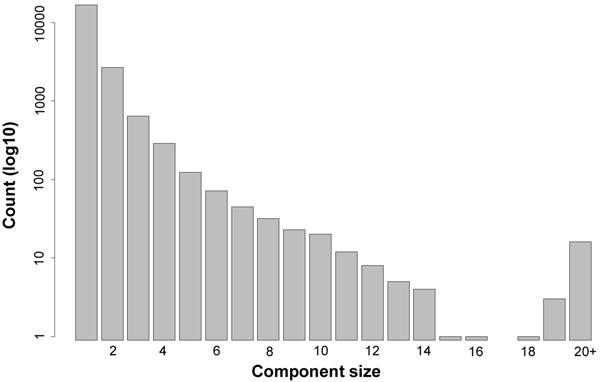
**Size of connected components in Arabidopsis genome, using 25 nucleotide reads.** The majority of the annotated Arabidopsis genes (22035 of 33282 genes) share 25-mers with at most one other gene. However, several genes, for example those that are part of extended gene families, share overlapping regions with a larger number of genes.

	This method partitioned the 33282 TAIR 8 gene models into 20660 connected components. The average size of a connected component was 1.6, indicating that most genes shared 25 nucleotide regions with 0 or 1 other genes. However, a number of larger components occurred as well. Figure 7 shows a histogram of component sizes. The largest component contained 5194 genes, while the next 10 largest components ranged in size from 26 to 58 genes. The full list of connected components is available in Additional file 8.

In order to characterize the types of genes contained in the largest components, we used the agriGO tool to analyze the associated GO terms. Several of the largest components appear to correspond to gene families and many were significantly enriched for terms such as “base pairing with RNA,” and “nucleic acid binding,” which are indicative of a functional significance for shared sequences. For example, the second largest component contains a family of genes encoding tRNAs for the amino acid, proline. The 58 members of this component share variations of the following 72 nucleotide sequence:

gggcatttggtctagtggtatgattctcgctttgggtgcgagaggtcccgagttcgattctcggaatgcccc

 In contrast, there were no significant terms for the very largest component and many of the edges in this 5194 gene “super-component” originated from various low complexity sequences. Table 2 contains descriptions of the 10 largest connected components, taken from the gene annotations available in TAIR.

**Table 2:** Putative function for genes in the 10 largest connected components in the Arabidopsis genome

Component	Size	Putative Function
1	5194	?
2	58	tRNA – PRO
3	57	tRNA – TYR
4	41	tRNA – SER
5	40	S-locus lectin protein kinase family
6	34	Receptor-like protein kinase
7	26	ECA1 gametogenesis related family protein
8	26	Transposable element gene; disease resistance protein
9	26	Transcription factors
10	26	Leucine-rich repeat protein kinase

## Discussion

We have developed a new method for using RNA-Seq data to quantify the alternatively spliced isoforms present in a mixture. Comparable existing methods for isoform quantification assume uniform sampling along targeted transcripts; however, real RNA-Seq datasets rarely exhibit uniform read sampling (e.g. Figure 1). Our simulations demonstrate that using the wrong read sampling distribution can lead to incorrect conclusions about the expression of isoforms. Accordingly, we have designed our method specifically to handle this problem. Some recent work has also suggested that read sampling distributions may include systematic biases that are transcript specific; for example, various aspects of RNA-Seq library preparation may introduce a dependence on the GC content of a given sequence and/or the terminal nucleotide ditags of the sampled sequence [22,23]. Our model, like other recent approaches to isoform quantification, does not take these sequence-specific biases into account. However, once these biases are better understood, they can potentially be incorporated into our model, for example, in the form of sequence-specific read start and length distributions. This is a topic that we are actively exploring.

“Multireads,” or reads that map to more than one gene, are another important problem for accurate isoform quantification. In this paper we have suggested two methods for estimation in the presence of multireads. One approach is to jointly estimate all genes that share overlapping sequences. An advantage of this approach, over the alternative which discards multireads (after making appropriate adjustments to the design matrix) is that we make use of all available information. However, in our connected components analysis, we have shown that the Arabidopsis genome includes gene families with 50 or more members. Although theoretically possible with our algorithm, a joint estimation of these transcripts, using all available information, is currently infeasible due to the increasing size of the design matrix.

One major limitation of our approach is that it assumes that all transcripts are known, yet the current state of transcriptome annotation is incomplete for most organisms. Because incorrect assumptions regarding potential transcripts in a mixture could lead to erroneous estimates, we are investigating ways to incorporate residual-based diagnostics into our model. These enhancements would serve to identify the presence of unknown “hidden” isoforms in a mixture and would complement isoform quantification with a mechanism for transcript discovery.

To demonstrate the practicality of our approach, we have applied it to two public Arabidopsis RNA-Seq experiments and the results reveal a high level of differential splicing between strains and treatments. In the first of the two example datasets, we identified 306 genes that exhibit differential splicing between two different Arabidopsis strains; in the second example dataset, we found 169 genes that were differentially spliced between drought-stressed and untreated Arabidopsis plants. In many of these cases, the predominant isoform differed between treatments, but we also identified a large number of genes that varied more subtly in isoform expression (see Figure 4). In both datasets, differential splicing and gene expression appear to be statistically independent events.

## Conclusions

 Next-generation high-throughput sequencing promises to revolutionize the study of the transcriptome. In this paper, we have introduced a new method for using RNA-Seq data to quantify the alternatively spliced isoforms present in a mixture. Our method, which can incorporate a non-uniform read sampling distribution, is flexible enough to accommodate a variety of sequencing technologies, including those that incorporate paired reads. We anticipate that the ability to reliably compute quantitative isoform expression values will help researchers to separate true alternative splicing events from spurious transcripts originating from single mis-spliced transcripts — a major problem in genome-wide alternative splicing studies. Based on recent studies [1,3] and our own observations derived from two independent Arabidopsis RNA-Seq experiments, isoform expression level changes frequently involve a continuum of isoform ratios, in addition to all-or-nothing expression patterns. Furthermore, in the datasets we have examined, isoform expression level changes appear to be independent of gene expression changes. This suggests the existence of a so far uninvestigated, dynamic layer of the transcriptome. For this reason, we believe that, in the future, researchers will prioritize genes for functional analysis based not only on observed changes in gene expression levels, but also on changes in alternative splicing.

## Competing interests

The authors declare that they have no competing interests.

## Authors' contributions

BEH implemented the method and analyzed the data. BEH and SH conceived of the method and study design, and collaborated to prepare the manuscript. All authors approved the final manuscript.

## Supplementary Material

Additional file 1 DiffAS_Lister.xls

Additional file 2 DiffAS_Mockler.xls

Additional file 3 Lister_Biological_Process.pdf

Additional file 4 Lister_Cellular_Component.pdf

Additional file 5 Lister_Molecular_Function.pdf

Additional file 6 Mockler_Biological_Process.pdf

Additional file 7 Mockler_Cellular_Component.pdf

Additional file 8 List of all connected components in a graph joining TAIR 8 genes that share 25-mer subsequences. ConnectedComponents.txt
