# Supplementary figures and images for: Towards reliable isoform quantification using RNA-SEQ data
Source: BMC Bioinformatics. 2010 Apr 29;11(Suppl 3):S6. doi: 10.1186/1471-2105-11-S3-S6 (PMC2863065; doi:10.1186/1471-2105-11-S3-S6)

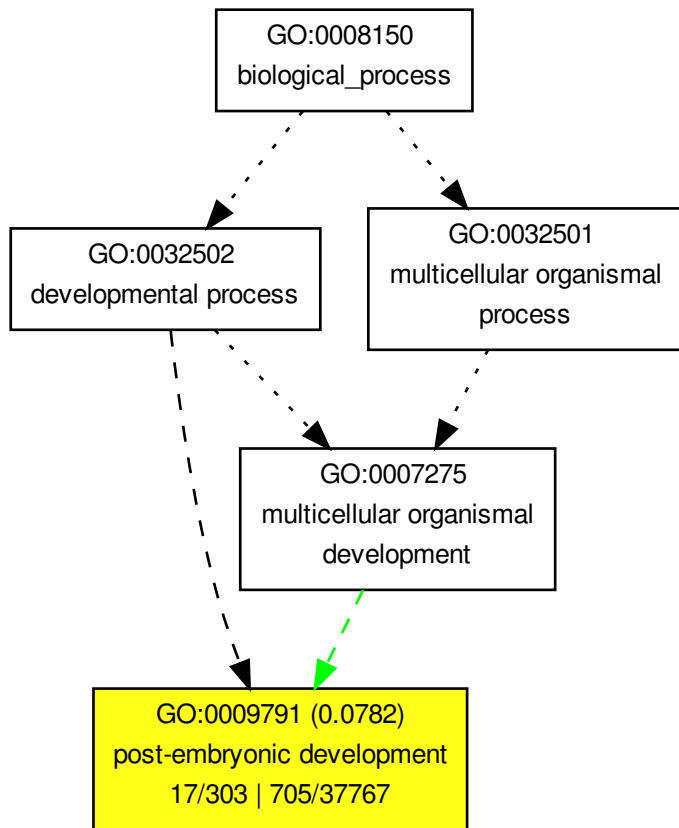

Supplement: Additional file 3 — Lister_Biological_Process.pdf [file 1471-2105-11-S3-S6-S3.pdf]

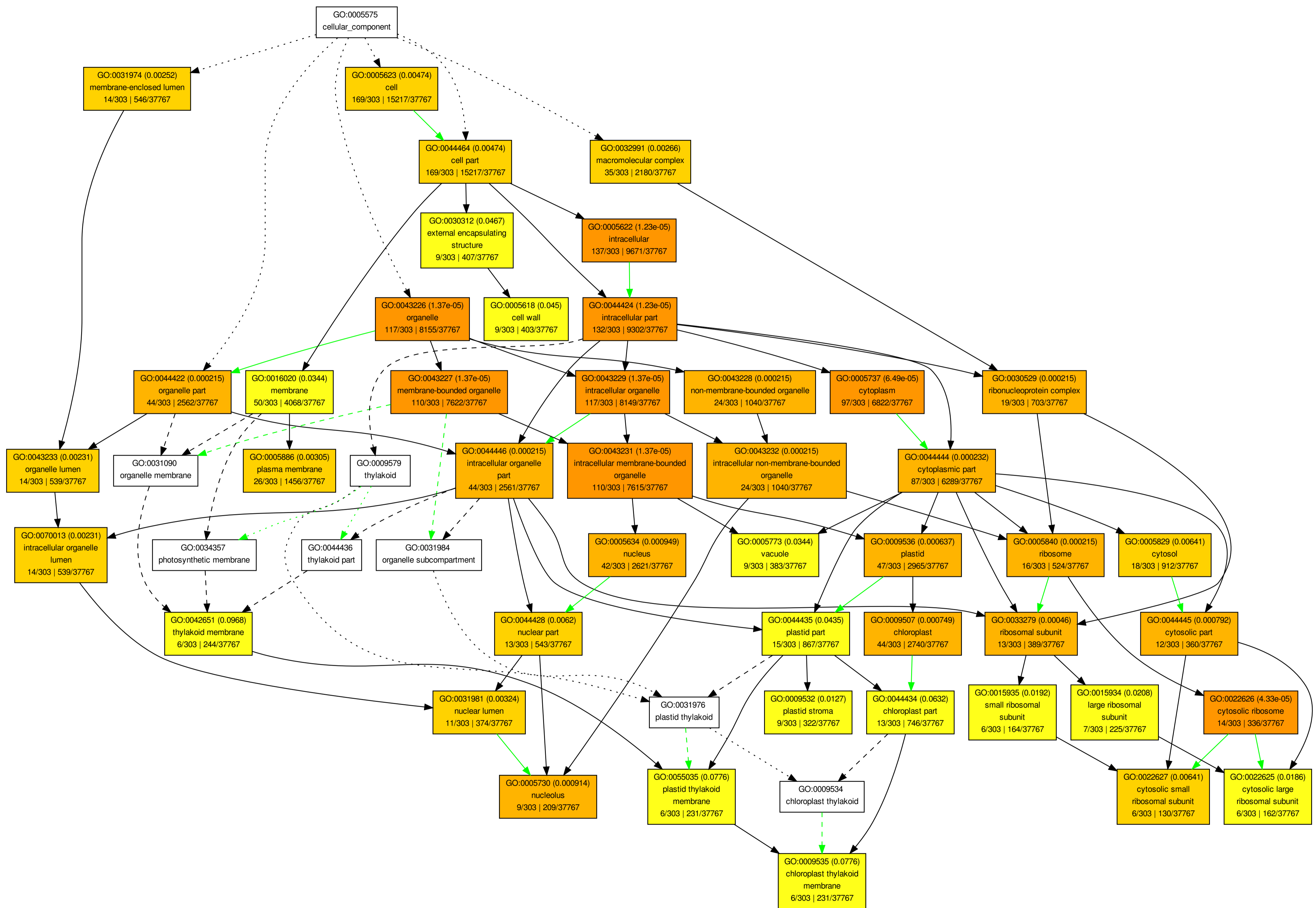

Supplement: Additional file 4 — Lister_Cellular_Component.pdf [file 1471-2105-11-S3-S6-S4.pdf]

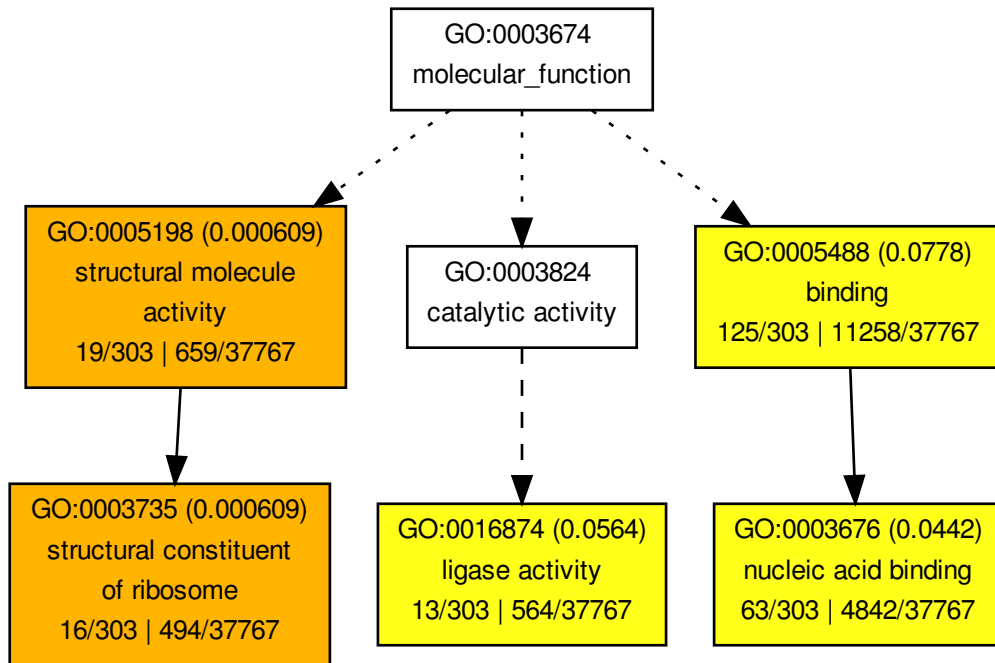

Supplement: Additional file 5 — Lister_Molecular_Function.pdf [file 1471-2105-11-S3-S6-S5.pdf]

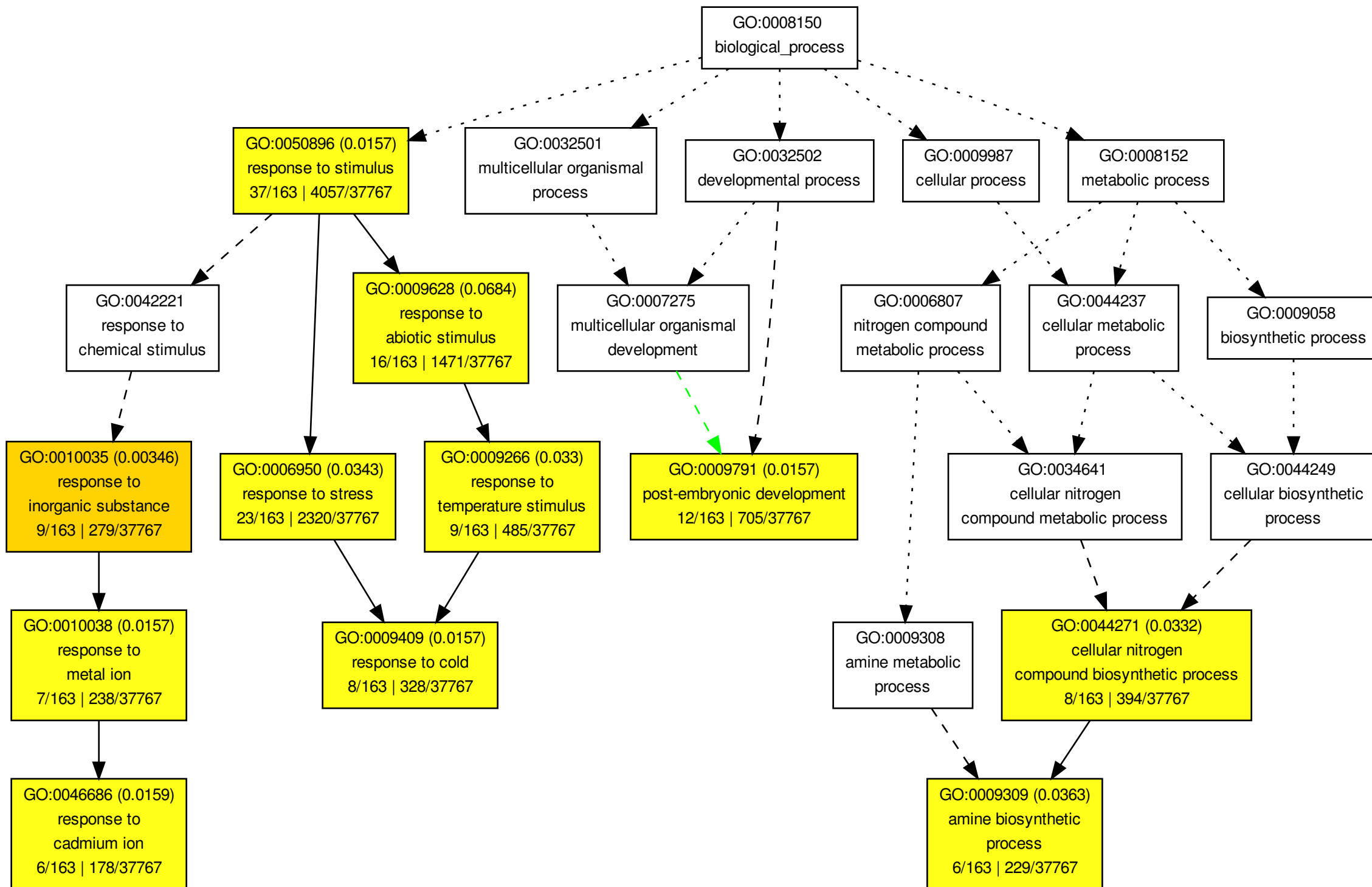

Supplement: Additional file 6 — Mockler_Biological_Process.pdf [file 1471-2105-11-S3-S6-S6.pdf]

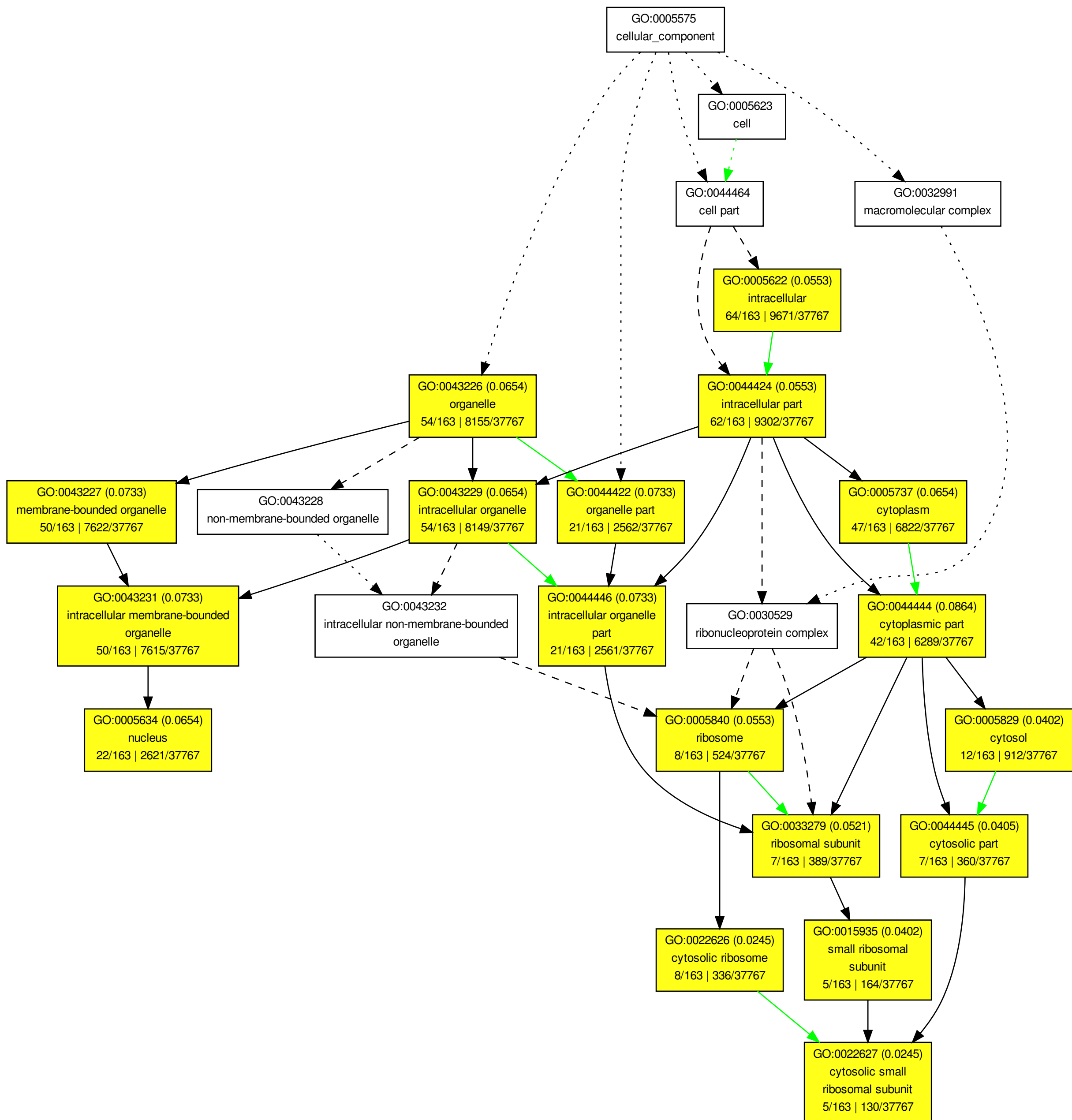

Supplement: Additional file 7 — Mockler_Cellular_Component.pdf [file 1471-2105-11-S3-S6-S7.pdf]
